# Contrasting molecular pathology of colorectal carcinoma in Egyptian and Western patients

**DOI:** 10.1054/bjoc.2001.1838

**Published:** 2001-09

**Authors:** A S Soliman, M L Bondy, S A El-Badawy, N Mokhtar, S Eissa, S Bayoumy, I A Seifeldin, P S Houlihan, J R Lukish, T Watanabe, A On On Chan, D Zhu, C I Amos, B Levin, S R Hamilton

**Affiliations:** Department of Epidemiology, The University of Texas M.D. Anderson Cancer Center, Houston, Texas, 77030, USA; The National Cancer Institute of Cairo University, Cairo, 11796, Egypt; Tanta Cancer Center and Faculty of Medicine, Tanta University, Tanta, Egypt; Division of Gastrointestinal-Liver Pathology, Department of Pathology, The Johns Hopkins University School of Medicine, Baltimore, Maryland, 21205, USA; Department of Pathology, Division of Pathology and Laboratory Medicine, The University of Texas M.D. Anderson Cancer Center, Houston, Texas, 77030, USA; Department of Surgery, National Naval Medical Center, Bethesda, Maryland, 20889, USA; Division of Cancer Prevention, The University of Texas M.D. Anderson Cancer Center, Houston, Texas, 77030, USA

**Keywords:** colorectal cancer, microsatellite instability, Egypt, schistosomiasis

## Abstract

Colorectal carcinoma is uncommon in Egypt, but a high proportion of cases occurs before age 40 years and in the rectum. We compared the molecular pathology of 59 representative Egyptian patients aged 10–72 to Western patients with sporadic, young-onset, or hereditary non-polyposis colorectal cancer syndrome (HNPCC)-associated carcinoma and found significant differences. Most Egyptian cancers were rectal (51%) and poorly differentiated (58%). High levels of microsatellite instability (MSI-H) were frequent (37%) and attributable in some cases (36%) to methylation of the promoter of the hMLH1 mismatch repair gene, but no MSI-H cancer had loss of hMSH2 mismatch repair gene product of the type seen with germline hMSH2 mutation in HNPCC. K-ras mutation was uncommon (11%). In subset analyses, high frequencies of MSI-H in rectal carcinomas (36%) and *p53* gene product overexpression in MSI-H cancers (50%) were found. MSI-H and K-ras mutation in Egyptians under age 40 were unusual (17% and 0%, respectively), and schistosomiasis was associated with MSI and K-ras mutation. Cluster analysis identified 2 groups: predominantly young men with poorly differentiated mucinous and signet-ring cell colorectal carcinoma lacking K-ras mutation; older patients who had well- or moderately differentiated adenocarcinoma often with MSI-H, K-ras mutation and schistosomiasis. Our findings show that the molecular pathology of colorectal cancer in older as well as younger Egyptians has unique differences from Western patients, and schistosomiasis influences the molecular pathogenesis of some tumours. © 2001 Cancer Research Campaignhttp://www.bjcancer.com


*British Journal of Cancer* (2001) **85**(7), 1037–1046
© 2001 Cancer Research Campaign
doi: 10.1054/ bjoc.2001.1838, available online at http://www.idealibrary.com on IDEAL®

http://www.bjcancer.com

### Contrasting molecular pathology of colorectal carcinoma in Egyptian and Western patients

**AS Soliman[1,2], ML Bondy[1], SA El-Badawy[2], N Mokhtar[2], S Eissa[2], S Bayoumy[3], IA Seifeldin[3], PS Houlihan[4,5], JR Lukish[6], T Watanabe[4], A On On Chan[5], D Zhu[1], CI Amos[1], B Levin[7] and SR Hamilton[4,5]**

[1]Department of Epidemiology, The University of Texas M.D. Anderson Cancer Center, Houston, Texas 77030, USA; [2]The National Cancer Institute of Cairo University, Cairo 11796, Egypt; [3]Tanta Cancer Center and Faculty of Medicine, Tanta University, Tanta, Egypt; [4]Division of Gastrointestinal-Liver Pathology, Department of Pathology, The Johns Hopkins University School of Medicine, Baltimore, Maryland 21205, USA; [5]Department of Pathology, Division of Pathology and Laboratory Medicine, The University of Texas M.D. Anderson Cancer Center, Houston, Texas 77030, USA; [6]Department of Surgery, National Naval Medical Center, Bethesda, Maryland 20889, USA; [7]Division of Cancer Prevention, The University of Texas M.D. Anderson Cancer Center, Houston, Texas 77030, USA

**Summary** Colorectal carcinoma is uncommon in Egypt, but a high proportion of cases occurs before age 40 years and in the rectum. We compared the molecular pathology of 59 representative Egyptian patients aged 10–72 to Western patients with sporadic, young-onset, or hereditary non-polyposis colorectal cancer syndrome (HNPCC)-associated carcinoma and found significant differences. Most Egyptian cancers were rectal (51%) and poorly differentiated (58%). High levels of microsatellite instability (MSI-H) were frequent (37%) and attributable in some cases (36%) to methylation of the promoter of the hMLH1 mismatch repair gene, but no MSI-H cancer had loss of hMSH2 mismatch repair gene product of the type seen with germline hMSH2 mutation in HNPCC. K-ras mutation was uncommon (11%). In subset analyses, high frequencies of MSI-H in rectal carcinomas (36%) and *p53* gene product overexpression in MSI-H cancers (50%) were found. MSI-H and K-ras mutation in Egyptians under age 40 were unusual (17% and 0%, respectively), and schistosomiasis was associated with MSI and K-ras mutation. Cluster analysis identified 2 groups: predominantly young men with poorly differentiated mucinous and signet-ring cell colorectal carcinoma lacking K-ras mutation; older patients who had well- or moderately differentiated adenocarcinoma often with MSI-H, K-ras mutation and schistosomiasis. Our findings show that the molecular pathology of colorectal cancer in older as well as younger Egyptians has unique differences from Western patients, and schistosomiasis influences the molecular pathogenesis of some tumours. © 2001 Cancer Research Campaign http://www.bjcancer.com

**Keywords**: colorectal cancer; microsatellite instability; Egypt; schistosomiasis

Colorectal cancer is the fourth most common cancer and the second most common cause of cancer deaths in the United States where the vast majority of cases occurs over age 60 years (Greenlee et al, 2000). In contrast, this disease is uncommon in developing countries (Magrath and Litvak, 1993), including Egypt where it is only the fifth most common cause of cancer deaths (Soliman et al, 1999). However, the percentage of young-onset colorectal cancer cases in Egyptians is strikingly high with more than one third of cases occurring under age 40 years, and the age-adjusted mortality rates in young Egyptians are likewise high (Soliman et al, 1997a, 1999). In addition, rectal cancer is frequent (Soliman et al, 1997a, 1999).

We have conducted a series of studies to characterize the distinctive distribution of age and anatomic location and to explore the possible risk factors for colorectal cancer in Egypt. We reported previously that the unusual disease pattern may be related to 2 major groups of risk factors: heavy environmental exposures such as organochlorine pesticides (Soliman et al, 1997b) and inherited genetic factors including familial aggregation (Soliman et al, 1998b), hereditary nonpolyposis colorectal cancer (HNPCC), and defective expression of mismatch repair genes (Soliman et al, 1998a). Schistosomiasis, particularly due to *S. japonicum*, has been implicated as an aetiologic factor in colorectal cancer (Chen et al, 1981; International Agency for Research on Cancer, 1994), and bilharziasis is common in Egypt (El-Khoby et al, 2000).

Recent advances in molecular biology have revealed numerous genetic alterations involved in colorectal tumorigenesis (Kinzler and Vogelstein, 1996; Gryfe et al, 1997; Ilyas et al, 1999). The development of most colorectal carcinomas begins with inactivation of the APC (adenomatous polyposis coli) suppressor gene/β-catenin pathway. Clonal accumulation of alterations then occurs, including activation of proto-oncogenes such as K-ras, and inactivation of additional suppressor genes. The genes commonly inactivated during progression include the *p53* gene on the short arm of chromosome 17 (Levine, 1997; Ding and Fisher, 1998). Mutated *p53* gene product fails to regulate normally a variety of genes regulated by wild-type *p53* (Cox, 1997). For many suppressor genes, including *p53*, inactivation of one allele is often caused by loss due to chromosomal instability of all or part of the chromosome where the gene resides, and allelic losses throughout the genome are common (Lengauer et al, 1998).

About 15% of sporadic colorectal cancers are distinguished by nucleotide insertions or deletions in numerous, intrinsically unstable repeated sequences in tumour DNA, termed microsatellite instability (MSI), also termed ubiquitous somatic mutations, DNA replication errors/RER, or nucleotide instability (Jiricny, 1998; Lengauer et al, 1998; Prolla, 1998). A workshop sponsored by the National

*Received 18 September 2000*
*Revised 22 February 2001*
*Accepted 28 February 2001*

*Correspondence to:* SR Hamilton

Cancer Institute in the United States developed criteria for classification of MSI in colorectal cancers (Boland et al, 1998). Tumours with high levels of MSI (MSI-H) are characteristic of hereditary nonpolyposis colorectal cancer syndrome (HNPCC), and MSI testing provides a sensitive diagnostic indicator of patients with the syndrome, although most MSI-H tumours in older patients are sporadic (Boland et al, 1998). MSI-H also occurs at high frequency in young patients with presumably sporadic colorectal cancer (Liu et al, 1995; Lukish et al, 1998; Chan et al, 1999).

HNPCC is caused by germline mutation of one of a group of DNA mismatch repair genes followed by somatic inactivation of the other allele (Lynch and Lynch, 2000). Abnormalities of the *hMLH1* gene or *hMSH2* gene are responsible for most HNPCC cases, and loss in tumours of immunohistochemical nuclear expression of the products of these genes often indicates which gene is mutated in the germline (Dietmaier et al, 1997; Fujiwara et al, 1998; Dieumegard et al, 2000; Muller et al, accepted). By contrast, sporadic MSI-H tumours usually occur due to somatic inactivation of both alleles of the hMLH1 mismatch repair gene, most commonly via transcriptional silencing which results from aberrant methylation of cytosines in the cytosine- and guanine-rich promoter region with loss of nuclear immunohistochemical expression of the gene product (Kane et al, 1997; Herman et al, 1998; Kuismanen et al, 2000). The genetic alterations which accumulate during progression of both hereditary and sporadic neoplasms characterized by MSI-H include mutations in genes with microsatellites within the coding region, such as the type II receptor for transforming growth factor β1 (TGFβ1RII) (Parsons et al, 1995; Fujiwara et al, 1998; Akhurst and Balmain, 1999; Abe and Masuda, 2000; Calin et al, 2000). Allelic deletions which are frequent in microsatellite-stable cancers are rare in MSI-H cancers (Fujiwara et al, 1998; Lengauer et al, 1998). Colorectal carcinomas with low levels of MSI (MSI-L) are uncommon and appear to arise through a pathway which differs from MSI-H and microsatellite-stable tumours, possibly involving hyperplastic polyps as a precursor (Iino et al, 1999; Jass et al, 1999).

The molecular pathology of colorectal carcinoma in Egypt has not been reported. We, therefore, studied the profile of pathological and molecular characteristics of colorectal cancers in patients from this country where adverse environmental exposures are high and genetic factors are known to be important (Soliman et al, 1997b, 1998a, 1998b). We analysed specimens from 59 Egyptian colorectal cancer cases for MSI status, methylation of the *hMLH1* promoter, loss of expression of *hMSH2* gene product, *K-ras* gene mutation in codons 12 and 13, and *p53* gene product overexpression of the type seen with *p53* gene mutation. We compared the molecular pathology to Western patients with sporadic, young-onset or HNPCC-associated colorectal cancer previously published from our laboratory (Jen et al, 1994a; Fujiwara et al, 1998; Lukish et al, 1998).

### MATERIALS AND METHODS

### Egyptian tumour specimens and patients

Paraffin-embedded blocks of unbuffered formalin-fixed colorectal carcinomas from 59 consecutive patients were included in this study. These patients participated in our previous study on familial aggregation of colorectal cancer in Egypt (Soliman et al, 1998b) and had their resection in 1996 and 1997 at the National Cancer Institute in Cairo ($n$ = 37) or Tanta Cancer Center in the mid Nile River Delta region ($n$ = 22). The study was approved by the institutional review boards at the MD Anderson Cancer Center and The Johns Hopkins Medical Institutions and was performed in accordance with filed institutional assurances. Information on demographics, residence, family history, tumour site and stage were retrieved from the medical records, pathology reports and our previous epidemiologic studies. Patients with familial adenomatous polyposis were excluded.

Patient age ranged from 10 to 72 years (mean 42.8 ± SD 15.1), and the series included 34 males (58%) and 25 females. All patients had stage II or stage III colorectal carcinoma. The residence of most patients was rural (78%). 27% of the patients were offspring of consanguineous marriages of first cousins. The study group was representative of our previous national study of 1608 colorectal cancer patients in Egypt (Soliman et al, 1997a), with no statistically significant differences in age, gender, residence or frequency of rectal cancer.

For each case, the histopathologic diagnosis of colorectal cancer was confirmed, and tumour differentiation and mucinous (colloid) and signet-ring cell histology were recorded based on criteria used in our previous studies (Jen et al, 1994a; Kim et al, 1994). All blocks of tumour tissue, nearby mucosa and distant surgical margins were serially sectioned (5 serials, 20 μm apart) for evaluation of schistosomal infection. Criteria included the presence of intact or calcified ova, active granulomas or worms. Speciation of schistosoma on the basis of morphology was not attempted.

### Comparison groups of Western patients

Previously published data for comparison with the Egyptian carcinomas were available from 3 groups of colorectal carcinomas analysed in our laboratory: (1) sporadic colorectal cancers ($n$ = 145) from stage II and III patients at The Johns Hopkins Hospital enrolled in a study of prognostic markers (Jen et al, 1994a); (2) young-onset colorectal cancers in patients under age 40 years ($n$ = 36) from a study at the National Naval Medical Center in Bethesda, Maryland (Lukish et al, 1998); and (3) HNPCC-associated colorectal cancers ($n$ = 39) from American, Canadian, and New Zealand patients in 20 families (Fujiwara et al, 1998) that met the International Collaborative Group criteria for HNPCC (Vasen et al, 1991) and/or had germline mutation of *hMSH2* or *hMLH1* mismatch repair genes (Wijnen et al, 1997; Genuardi et al, 1998).

### Microdissection and DNA extraction

For all 59 Egyptian tumours, areas of carcinoma and non-neoplastic tissue were microdissected from numbered tissue sections. Cellular tumour that contained few stromal and inflammatory cells was selected. Multiple areas with high cellularity from each slide were combined to enhance the detection of clonal abnormalities. DNA was extracted as in our previous studies (Jen et al, 1994a; Moskaluk and Kern, 1997; Fujiwara et al, 1998; Lukish et al, 1998).

### Assay for microsatellite instability (MSI)

All molecular and immunohistochemical analyses were done without access to clinical data. MSI was evaluated by polymerase chain reaction (PCR) amplification of 8 non-coding polymorphic dinucleotide repeat sequences, 2 non-coding rarely polymorphic polyadenine tracts (BAT26 and BAT25), and 1 coding rarely polymorphic

 *© 2001 Cancer Research Campaign*

polyadenine tract in the transforming growth factor beta 1 type II receptor gene (*TGFβ1RII*), as in our previous studies (Jen et al, 1994a, 1994b; Parsons et al, 1995; Fujiwara et al, 1998). The 8 dinucleotide repeat sequences included 5 on the long arm of chromosome 18 (D18S69, D18S64, D18S55, D18S61, and D18S58 from centromere to telemere), 2 on the long arm of chromosome 5 (D5S107 and D5S346), and 1 on the short arm of chromosome 2 (D2S123).

The normal alleles were represented by 1 (non-polymorphic) or 2 (polymorphic) major bands accompanied by a few minor bands. The mobility shift of PCR products from tumour DNA as compared with corresponding non-neoplastic tissue DNA was determined for each marker independently. Each tumour was classified from the aggregated marker results as: (1) high levels of MSI (MSI-H) if at least 2 markers representing at least 30% of informative dinucleotide or mononucleotide markers were shifted (Boland et al, 1998) and/or mutation was present in TGFβ1RII (Dietmaier et al, 1997; Perucho, 1999); (2) low levels of MSI (MSI-L) if only 1 marker among a minimum of 5 dinucleotide and mononucleotide markers was shifted; (3) microsatellite-stable if no marker among a minimum of 5 informative markers was shifted; (4) indeterminate for MSI if 3 or fewer markers without shift were informative.

### Assay for methylation of *hMLH1* promoter

Somatic and germline mechanisms leading to MSI were evaluated. Somatic methylation of the promoter of the *hMLH1* mismatch repair gene is associated with transcriptional silencing of this gene and resultant MSI-H. The methylation status of *hMLH1* in Egyptian carcinomas with known MSI status was determined by bisulfite treatment of DNA followed by methylation-specific polymerase chain reaction (MSP-PCR) as described (Herman et al, 1996) with modification of primers for methylated and unmethylated alleles and cycling conditions. Detailed protocols are at the website www.mdanderson.org/leukemia/methylation/. In brief, 2 μg of genomic DNA from microdissected tumour were denatured with sodium hydroxide, incubated with sodium bisulfite, purified with DNA Cleanup Kit (Promega), incubated with sodium hydroxide, precipitated with ammonium acetate and ethanol, washed with ethanol and resuspended in distilled water. The primers were: methylated allele, sense primer: 5′-GATAGCGATTTTTAACGC-3′; methylated allele, antisense primer: 5′-TCTATAAATTACTAAATCTCTTCG-3′; unmethylated allele, sense primer: 5′-AGAGTGGATAGTGATTTTTAATG-T-3′ unmethylated allele, antisense primer: 5′-ACTCTATAAAT-TACTAAATCTCTTCA-3′. Cycling conditions were 95˚C for 10 minutes and 40 cycles of 95˚C for 30 seconds and 53˚C for 45 seconds. PCR products for methylated and unmethylated reactions were electrophoresced on acrylamide gels and visualized by ethidium bromide staining. Gel photographs were digitized using a BioRad imager and evaluated by densitometry using the manufacturer’s software. The results were expressed as percentage of methylation by comparing the density of the methylated band relative to the sum of the methylated and unmethylated bands. Because the sensitivity of MSP-PCR can lead to overestimation of low levels of methylation, only loci showing ≥ 10% methylation were considered methylated.

### Immunostaining for *hMSH2* and *hMLH1* gene products

Loss of nuclear expression of *hMSH2* gene product is strongly associated with germline mutation of the *hMSH2* gene in patients with HNPCC (Dietmaier et al, 1997; Fujiwara et al, 1998; Dieumegard et al, 2000; Muller et al, accepted). Loss of *hMLH1* gene product can occur with germline mutation of the gene in HNPCC patients (Muller et al, accepted) and with transcriptional silencing by somatic methylation (Kane et al, 1997; Herman et al, 1998; Kuismanen et al, 2000). The subset of Egyptian carcinomas previously characterized for MSI status was evaluated by immunohistochemistry after antigen retrieval with citrate buffer. hMSH2 antibody (clone FE11, Oncogene Research Products, Boston, MA) was used at 1:100 dilution and hMLH1 antibody (clone G18–15, Pharmingen, San Diego, CA) at 1:30 dilution in the TechMate 1000 automated staining system (Ventana/BioTek Solutions, Tucson, AZ), similar to our previous studies (Fujiwara et al, 1998). Each stained slide was evaluated for positive-control staining of nuclei in epithelial cells at the bases of colorectal crypts and/or germinal centres of lymphoid nodules. Tumour cell nuclei in the immediate vicinity of internal positive controls were then interpreted for the presence or absence of nuclear staining. As in our and other previous studies (Fujiwara et al, 1998; Muller et al, accepted), hMLH1 staining was inconsistent and therefore not evaluated further.

### Assay for *K-ras* proto-oncogene mutations

All possible mutations in codons 12 and 13 of the *K-ras* proto-oncogene were determined as in our previous studies (Jen et al, 1994b; Nucci et al, 1997). Exon 1 was amplified, and the resulting PCR product from each tumour was sequenced using the SequiTherm EXCEL II™ DNA sequencing kit (Epicentre Technologies, Madison, WI).

### Immunostaining for *p53* gene product overexpression and mutation analysis

Detection of *p53* gene product overexpression by immunohistochemistry was used as an indicator of *p53* gene mutation. This method provides about 75% overall accuracy in colorectal neoplasms owing to the prolonged half life of most mutant proteins in such tumours (Baas et al, 1994). DO7 mouse monoclonal antibody against *p53* (Dako, Carpinteria, CA) was used at 1:100 dilution in the TechMate 1000 automated staining system (Ventana/BioTek Solutions, Tucson, AZ), as in our previous studies (Moskaluk et al, 1996). *p53* labelling index of > 40% in a discrete region of or entire tumour was considered positive. Exons 5 through 9 of the *p53* gene were amplifiable and sequenced as in our previous studies (Moskaluk et al, 1996) in 3 MSI-H cancers with *p53* gene product overexpression and high-quality DNA.

### Statistical analysis

Differences in frequencies between the Egyptian group and the Western groups and between subsets within the Egyptian group were evaluated by simple contingency table analysis (Fisher’s exact probability test) using the SAS statistical package (Statistical Analysis System User’s Guide, 1990) and true Epistat. *P* values are two-sided.

Cluster analysis was performed to identify subgroups of Egyptian patients who had similar demographic, pathologic and molecular attributes (Anderberg, 1973). Variables that were included in this analysis were age (categorized into 2 groups younger or older than 40 years), sex, residence (urban versus rural), schistosomiasis (presence or absence of histopathologic

© 2001 Cancer Research Campaign 

evidence), tumour site (colon versus rectum), tumour histology (4 variables defined by glandular, mucinous, signet-ring cell or other histopathologic type), MSI status (one variable contrasting MSI-H versus MSI-L and microsatellite-stable cancers, and another variable contrasting MSI-L versus MSI-H and microsatellite-stable tumours), *K-ras* gene mutation, and *p53* gene product overexpression. We first re-scaled the data as a distance measure using one minus the Jaccard coefficient (Anderberg, 1973). We used the average linkage cluster analysis method and chose the number of clusters by evaluating the pseudo $t^2$. In order to create clusters in a parsimonious manner, we performed the cluster analysis, evaluated the resulting clusters to identify any variables that did not vary significantly among the clusters, and then removed those variables.

### RESULTS

The Egyptian patients were young, with ages ranging from 10–72 years and with 44% of patients under age 40 (Table 1). The pathologic features of the Egyptian colorectal cancers were unusual as compared to Western patients. About half of the 59 Egyptian carcinomas (51%) were located in the rectum (Table 1), as contrasted with less than 25% in all 3 Western groups ($P$ = 0.009 to < 0.0001, Table 2). Most Egyptian tumours (58%) were poorly differentiated, and about a third (37%) were mucinous (Figure 1); by contrast, among Western cases poorly differentiated tumours were frequent only in HNPCC patients (Table 2). Tumour histopathology differed between younger and older Egyptians as well: a significantly larger proportion of tumours from patients under 40 years of age were mucinous and poorly differentiated ($P$ = 0.03 and $P$ = 0.009, respectively, Table 3). Schistosomiasis was evident in tumour and/or adjacent tissue of 32% of patients and was unrelated to clinical and other pathologic features.

Consistent with the unusual tumour pathology, the molecular characteristics of the Egyptian cancers also differed from Western patients studied in our laboratory (Table 2). MSI-H occurred in 37% of Egyptian carcinomas. Among the 10 MSI-H cases evaluated with dinucleotide and mononucleotide markers, the percentage of altered markers ranged from 33% to 100% with a mean of 61 ± 8% (standard error) and a median of 47%. 8 of these 10 MSI-H cancers had informative mononucleotide markers, and all had at least 1 shifted. 4 MSI-H cases were evaluated with mononucleotide markers only. 3 had at least 2 mononucleotide markers shifted, and the fourth had a

**Table 1** Clinical characteristics of 59 colorectal cancer patients in Egypt

| Characteristic | Frequency in % (No.) |
|---|---|
| **Age (years)** | |
| < 40 | 44 (26) |
| 40 + | 56 (33) |
| Gender | |
| Male | 58 (34) |
| Female | 42 (25) |
| Residence | |
| Rural | 78 (46) |
| Urban | 22 (13) |
| Tumour site | |
| Ascending colon | 25 (15) |
| Transverse colon | 5 (3) |
| Descending colon | 15 (9) |
| Rectosigmoid colon | 3 (2) |
| Rectum | 51 (30) |

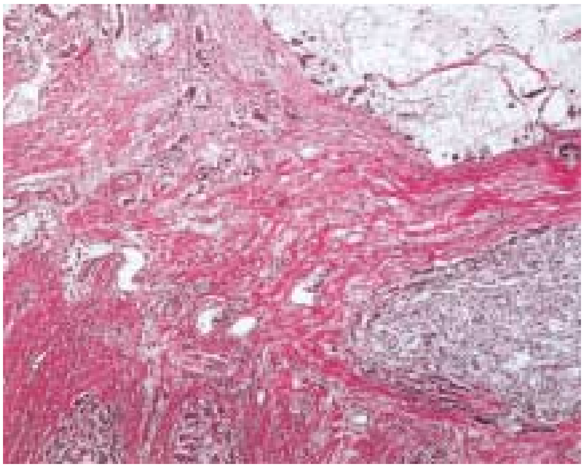

**Figure 1** Histopathology of Egyptian rectal carcinoma. The tumour has mucinous adenocarcinoma with malignant epithelium within pools of mucin (upper right quadrant) and poorly differentiated adenocarcinoma with solid areas (lower right quadrant) and rudimentary gland formation (left quadrants)

mutation in *TGFβ1RII*. MSI-H was explained by methylation of the promoter of the *hMLH1* mismatch repair gene in 36% of the cancers (Table 4, Figure 2), but no loss of nuclear *hMSH2* mismatch repair gene product indicative of germline *hMSH2* mutation was found (Table 4, Figure 3). MSI-L was found in 16% of cancers, based on shift of 1 of 5 to 1 of 11 informative dinucleotide and mononucleotide markers.

The frequency of MSI-H tumours in the Egyptian group as a whole was higher than in the Western sporadic group (37% vs. 13%, $P$ = 0.002) but much lower than the HNPCC group (95%, $P$ < 0.0001) and not significantly different from the young-onset group (47%). However, the Egyptian cases were heterogeneous, as MSI-H cancer was somewhat more commonly seen in older than younger Egyptian patients (17% vs 46% $P$ = 0.15, Table 3), and in those from urban areas (50% vs. 22%, $P$ = 0.002). A family history of a first-degree relative with colorectal cancer was present in only 1 patient with an MSI-H cancer, who was in the older age group (48 years old). The Egyptian patients had by far the highest proportion of MSI-H tumours located in the rectum as compared to the 3 groups of Western patients (36% vs. 0% to 6%, Table 2).

*K-ras* mutation was unusual in Egyptian colorectal cancers as compared with Western patients (11% vs. 28–48%, $P$ = 0.05 to 0.0004, Table 2 and Figure 2). There was a trend toward higher prevalence of *K-ras* mutation in older than younger Egyptian patients (17% vs 0%, $P$ = 0.14, Table 3). *p53* overexpression was somewhat more common in cancers of younger Egyptian patients compared with older Egyptian patients (57% vs. 39%, Table 3 and Figure 4), but the difference was not statistically significant.

When profiles of multiple alterations were considered, the frequency of MSI-H cancers with *K-ras* mutation was comparable in the Egyptian and Western groups, but the frequency of MSI-H tumours with *p53* overexpression of the type seen with *p53* gene mutation was markedly higher among Egyptians (50% vs 0% in the sporadic group and 5% in the HNPCC group, Table 2). Sequencing of exons 5–9 of the *p53* gene in 3 available MSI-H Egyptian cancers with *p53* gene product overexpression confirmed a mutation in 2 (T to A transversion in codon 137, nucleotide 410 of exon 5, CTG to CAG, leu to gln; G to A transition in codon 238, nucleotide 713 of exon 7, TGT to TAT, cys to tyr). These mutations have been reported previously in colorectal and other tumours (Hainaut et al, 1998).

 *© 2001 Cancer Research Campaign*

**Table 2** Comparison of pathology and molecular pathology in colorectal cancers of patients in Egypt and 3 Western groups

| | Egyptians | Westerners | | |
|---|---|---|---|---|
| | **%<br>(95% CI)<br>*n* positive/*n* examined** | **Sporadic<br>%<br>(95% CI)<br>*n* positive/*n* examined<br>*P* value** | **Young (≤ 40 yrs)<br>%<br>(95% CI)<br>*n* positive/*n* examined<br>*P* value** | **HNPCC<br>%<br>(95% CI)<br>*n* positive/*n* examined<br>*P* value** |
| **Pathology** | | | | |
| Rectal cancer | 51<br>(38–64)<br>30/59 | 19<br>(13–26)<br>27/145<br>< 0.0001 | 22<br>(10–39)<br>8/36<br>0.009 | 3<br>3 (0–13)<br>1/39<br>< 0.0001 |
| Poor differentiation | 58<br>(44–70)<br>34/59 | 15<br>(10–22)<br>22/145<br>< 0.0001 | 22<br>(10–39)<br>8/36<br>0.001 | 56<br>(40–72)<br>22/39<br>0.99 |
| Mucinous type | 37<br>(25–51)<br>22/59 | 11<br>(6–17)<br>16/145<br>< 0.0001 | 14<br>(5–30)<br>5/36<br>0.02 | Data not available |
| **Molecular pathology** | | | | |
| MSI-H | 37<br>(22–54)<br>14/38 | 13<br>(8–20)<br>18/137<br>0.002 | 47<br>(30–65)<br>17/36<br>0.48 | 95<br>(83–99)<br>37/39<br>< 0.0001 |
| K-ras mutation in codon 12 or 13 | 11<br>(4–23)<br>5/47 | 28<br>(21–37)<br>41/145<br>0.02 | 48<br>(30–67)<br>15/31<br>0.0004 | 28<br>(15–45)<br>11/39<br>0.05 |
| p53 gene product overexpression | 46<br>(33–60)<br>26/56 | 36<br>(24–50)<br>21/58<br>0.34 | Not done | 8<br>(2–21)<br>3/39<br>< 0.0001 |
| MSI-H in rectal cancers | 36<br>(13–65)<br>5/14 | 0<br>0 (0–19)<br>0/18<br>0.01 | 6<br>(0–29)<br>1/17<br>0.07 | 3<br>(0–14)<br>1/37<br>0.004 |
| MSI-H and K-ras mutation | 23<br>(5–54)<br>3/13 | 18<br>(4–43)<br>3/17<br>0.99 | 33<br>(13–59)<br>6/18<br>0.70 | 31<br>(17–49)<br>11/35<br>0.73 |
| MSI-H and p53 overexpression | 50<br>(23–77)<br>7/14 | 0<br>(0–19)<br>0/18<br>0.001 | Not done | 5<br>(1–18)<br>2/37<br>0.0007 |

Schistosomiasis was unequally distributed relative to MSI and *K-ras* gene mutation status. Ova were identified in 50% (8/16) of the patients with MSI-H cancers and in 67% (4/6) of the MSI-L patients, but in only 22% (7/32) of the patients with microsatellite-stable cancers ($P = 0.002$). Schistosomiasis was also more frequent among patients whose tumours showed *K-ras* gene mutation (80% of the 5 patients whose tumour had *K-ras* mutation vs. 31% of the 42 patients whose tumour was negative for *K-ras* mutation, $P = 0.05$).

Complete data including *K-ras* mutation analysis were available for 46 patients. We therefore performed cluster analyses in this subset of patients including and excluding those with *K-ras* mutation data, and the results were qualitatively similar. Initial analysis showed no effect of *p53* and tumour site in differentiating the clusters, so these variables were dropped from further analysis. The pseudo $t^2$ statistics for 1 versus 2 clusters were 19.3 and 8.5, suggesting that a model with 2 clusters would adequately describe the data. The first cluster was composed predominantly of young men with poorly differentiated carcinomas including mucinous or signet-ring cell histologies (Table 5). No patient in this cluster had *K-ras* mutation. MSI-H was infrequent, although MSI-L was evident in about 20% of patients, especially those with schistosomiasis. By contrast, the second cluster included predominantly older patients with well- or moderately differentiated gland-forming adenocarcinoma and a higher frequency of MSI-H, *K-ras* mutation and schistosomiasis than the first

*© 2001 Cancer Research Campaign* 

**Table 3** Age-related differences in pathology and molecular pathology in colorectal cancers of Egyptian patients

| Tumour characteristic | Age (years) < 40 % | < 40 Fraction | 40+ % | 40+ Fraction | *P* |
|---|---|---|---|---|---|
| Differentiation | | | | | |
| Poor | 77 | 20/26 | 42 | 14/33 | 0.009 |
| Moderate or well | 23 | 6/26 | 58 | 19/33 | |
| Mucinous carcinoma | 54 | 14/26 | 24 | 8/33 | 0.03 |
| MSI-H carcinoma | 17 | 2/12 | 46 | 12/26 | 0.15 |
| K-ras gene mutation | 0 | 0/18 | 17 | 5/29 | 0.14 |
| p53 overexpression | 57 | 13/23 | 39 | 13/33 | 0.28 |

**Table 4** Evaluation of colorectal cancers from Egyptian patients for mechanisms producing microsatellite instability

| Mechanism | Microsatellite instability status: MSI-H % (95% CI) *n* positive/*n* examined | MSI-L % (95% CI) *n* positive/*n* examined | MS Stable % (95% CI) *n* positive/*n* examined |
|---|---|---|---|
| Loss of nuclear hMSH2 gene product (germline) | 0<br>(0–31)<br>0/10 | 0<br>(0–60)<br>0/4 | 0<br>(0–23)<br>0/14 |
| Methylation of hMLH1 promoter (somatic) | 36<br>11–69<br>4/11 | 0<br>0–52<br>0/5 | 0<br>0–19<br>0/18 |

cluster. This group had characteristics more similar to colorectal cancer in Western countries, except for the presence of schistosomiasis.

### DISCUSSION

We report here that the molecular pathology of colorectal carcinoma in Egypt differs from Western patients, and between younger and older Egyptians. These findings reinforce the conclusion from our previous epidemiologic studies (Soliman et al, 1997a, 1999) that the pathogenesis of colorectal carcinoma in Egypt has unique features. Although Arabic countries share some cultural background and environmental exposures (Temtamy et al, 1994; Al-Saleh et al, 1998), rates of young-onset disease are higher in Egypt than in Jordan (Al-Jaberi et al, 1997), Lebanon (Adib et al, 1998), Algeria (Parkin et al, 1992), and Saudi Arabia (Isbister, 1992).

Occurrence of colorectal carcinoma at young age in Egypt could reflect the presence of clinically inapparent inherited syndromes. We excluded patients with history of or morphologic evidence of familial adenomatous polyposis. HNPCC is unlikely because tumours with loss of *hMSH2* gene product of the type seen with germline *hMHS2* mutation were not identified in our study. MSI-H cancers in young patients suggestive of HNPCC were rare, and the frequency of family history of colorectal or other HNPCC-associated cancers was low. In addition, some of the MSI-H cancers were explained by methylation of the *hMLH1* promoter. There is a high prevalence of consanguinity in Egypt, reflected in our study group, because of the tradition of interfamilial marriages (Temtamy et al, 1994), and this cultural characteristic could contribute to non-syndromic inherited predisposition.

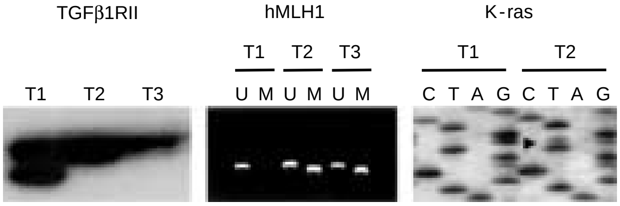

**Figure 2** Assays for mutation in gene for transforming growth factor beta1 type II receptor (TGFβ1RII, left panel), methylation of the promoter of the hMLH1 mismatch repair gene (centre panel), and mutation in codons 12 and 13 of the K-ras proto-oncogene (right panel) in Egyptian colorectal carcinomas. Polymerase chain reaction (PCR) amplification of the polyadenine tract of the TGFβ1RII gene for 3 tumours (lanes T1–T3) shows wild-type alleles (arrow) in all 3 and a mutated allele with a 2 basepair deletion in lane T1 (arrowhead). Methylation-specific PCR using primers specific for methylated (M) and unmethylated (U) alleles in bisulfite-treated DNA from 3 tumours shows abnormal methylated alleles in lanes T2 and T3 but not T1. Sequencing of PCR products after amplification of K-ras shows a G to T transversion in the second basepair of codon 12 (arrowhead) in lanes of T2

Poorly differentiated carcinomas with mucinous and signet-ring cell histology occurred in our first cluster (Table 5) of young Egyptian patients with low frequency of schistosomiasis. Studies in Western countries with high risk of colorectal cancer and in low-risk populations such as the black populations of South Africa and East Africa have also shown increased frequency of mucinous histology and rectal cancer in young patients (Bedikian et al, 1981; Adloff et al, 1986; Peters et al, 1989; Heys et al, 1994; Steele, 1994; Rubin et al, 1998; Boytchev et al, 1999). Epidemiologic studies indicate that tumour site may be related to specific risk factors affecting an anatomical segment of the colorectum (Williams et al, 1977; Weisburger et al, 1980; Peters et al, 1989). We found, however, that the rectal predominance in Egyptian cases occurred in both younger and older patients and was unrelated to urban/rural residence, which is associated with very different environmental exposures.

Both microsatellite-stable and MSI-H carcinomas in our series had unusual molecular genetic characteristics in comparison to Western tumours. Mutation of the *K-ras* proto-oncogene was uncommon in Egyptian colorectal cancers in general, in contrast to Western cases (Gryfe et al, 1997; Ilyas et al, 1999), and was not found in any patient under age 40 years in our study. Low frequency

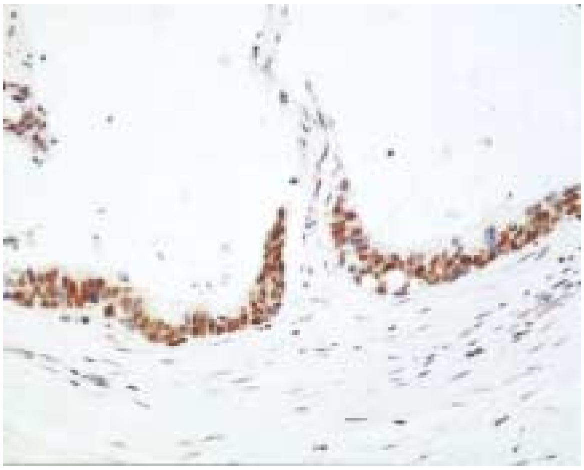

**Figure 3** Immunohistochemistry of hMSH2 mismatch repair gene product in Egyptian rectal cancer. The mucinous carcinoma retained nuclear expression of hMSH2

*© 2001 Cancer Research Campaign*

**Table 5** Distribution of characteristics among clusters of Egyptian patients with colorectal carcinoma

| | Prevalence of characteristic (%) | |
|---|---|---|
| **Characteristic**[a] | **First cluster (*n* = 17 patients)** | **Second cluster (*n* = 29 patients)** |
| Demographics | | |
| Age < 40 years | 77 | 17 |
| Male sex | 71 | 41 |
| Schistosomiasis | 18 | 48 |
| Tumour histology | | |
| Poor differentiation | 100 | 45 |
| Glandular type | 53 | 79 |
| Mucinous type | 71 | 21 |
| Signet-ring cell type | 88 | 4 |
| Molecular findings in tumour | | |
| MSI-H | 12 | 38 |
| MSI-L | 18 | 7 |
| K-ras gene mutation | 0 | 17 |

[a]Characteristics included in the cluster analysis which did not differentiate among the clusters were anatomic site (colon vs rectum), residence (urban vs rural) and *p53* gene product overexpression.

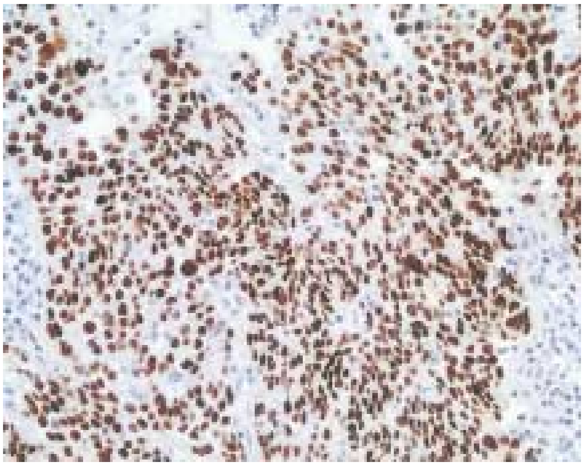

**Figure 4** Immunohistochemistry of *p53* gene product in Egyptian colon cancer. The poorly differentiated carcinoma has nuclear overexpression of *p53* gene product of the type seen with *p53* gene mutation of *K-ras* mutation has also been found previously in colorectal carcinomas in the low-incidence population in Singapore, with increasing mutation frequency associated with increasing incidence in the population in recent years (Tang et al, 1999). *K-ras* mutation is also uncommon in rectal carcinomas of Finnish women (Servomaa et al, 2000). MSI-H cancers are rarely located in the rectum in Western populations (Boland et al, 1998; Lukish et al, 1998), but fully half of Egyptian MSI-H cases were at that site. MSI-H carcinomas in our Egyptian series had frequent overexpression of *p53* gene product of the type seen with *p53* gene mutation, and sequencing confirmed the presence of a *p53* gene mutation in 2 of 3 informative cases. By contrast, *p53* overexpression is uncommon in MSI-H cancers in a large number of Western series, including our own (Ionov et al, 1993; Konishi et al, 1996; Breivik et al, 1997; Losi et al, 1997; Olschwang et al, 1997; Ruschoff et al, 1997; Forster et al, 1998; Lukish et al, 1998; Yagi et al, 1998).

Schistosomiasis with *S. japonicum* is associated with risk of colorectal cancer in studies correlating incidence, prevalence and/or mortality rates of the 2 diseases and in case–control studies done mainly in China and Japan (Chen et al, 1981; International Agency for Research on Cancer, 1994). Studies in Egypt showed an average prevalence of *S. haematobium* of 7.8% in Upper Egypt and 36.4% for *S. mansoni* in Lower Egypt in the respective endemic areas (El-Khoby et al, 2000). Infection rates were higher in children and adolescents and in males. 21% of young children in the Nile Delta region had schistosomiasis in another study (Curtale et al, 1998). We identified by histopathology a prevalence of 32% in our resection specimens of colorectal carcinoma from 2 cancer centres in Cairo and Lower Egypt, a rate comparable to the general population. We also found associations between molecular characteristics of colorectal carcinomas and infection. Although our patient numbers are small, our results suggest that schistosomiasis is associated with microsatellite instability and with *K-ras* mutation in Egyptian colorectal carcinomas (cluster 2 in Table 5), favouring schistosomiasis as a factor influencing pathogenesis in some cancers. This finding also indicates different molecular mechanisms for these genetic changes than in Western patients. A previous study has related the type of *p53* mutation in rectal cancer to schistosomiasis with *S. japonicum* (Zhang et al, 1998), but the mechanisms resulting in the differing profiles of accumulated genetic alterations in Egyptian and Western cancers remain unexplained.

The morphologic precursors to colorectal carcinoma in Egypt have not been studied extensively. Flat adenomas have been characterized as an important precursor to colorectal carcinoma (Rembacken et al, 2000). These lesions have a low frequency of *K-ras* gene mutation (Jass, 1995), as does dysplasia complicating chronic inflammatory bowel disease in the form of ulcerative colitis and Crohn colitis, in which MSI-H is relatively common (Umetani et al, 1999). Our finding that *K-ras* gene mutation is infrequent in Egyptian colorectal carcinomas unassociated with schistosomiasis raises the possibility that the flat adenoma could be an important precursor.

Westernization of Egypt is occurring as the country develops and is affecting young people first because they are more likely to change lifestyle than older people (Rockhill and Giovannucci, 1999). The remarkable differences in molecular pathology as compared to Western patients could result from inherent differences

© 2001 Cancer Research Campaign 

in sensitivity and molecular responses to Western lifestyle in Egyptians (Rockhill and Giovannucci, 1999). Schistosomiasis appears to be important in colorectal carcinogenesis in some cases. The outcome of the search for germline predispositions, environmental factors and gene-environment interactions that are responsible for our observations will impact strategies for prevention as well as advance knowledge of the pathways involved in the molecular pathogenesis of colorectal carcinoma.

### ACKNOWLEDGEMENTS

We appreciate the assistance with photomicrography from Dr Victor Prieto and David Sanders, editorial comments of Jude Richard in the Department of Scientific Publications, and the secretarial support of Joyce Brown in the Department of Epidemiology and Cheryl Willis in the Division of Pathology and Laboratory Medicine at MD Anderson Cancer Center. This work was supported in part by grant 980038 from the Middle East Cancer Consortium, a pilot grant from the Center for Research on Environmental Disease of NIEHS, a fellowship from the Cancer Research Foundation of America, the Ellen F. Knisely Distinguished Professorship, the Betty Marcus Chair in Cancer Prevention, the Caroline Law Fund, and NIH grants CA62924 and ES09912.

### REFERENCES

Abe Y and Masuda H (2000) Genetic alterations of sporadic colorectal cancer with microsatellite instability, especially characteristics of primary multiple colorectal cancers. *J Surg Oncol* **74**: 249–256

Adib SM, Mufarrij AA, Shamseddine AI, Kahwaji SG, Issa P and El-Saghir NS (1998) Cancer in Lebanon: an epidemiological review of the American University of Beirut Medical Center Tumor Registry (1983–1994). *Annal Epidemiol* **8**: 46–51

Adloff M, Arnaud J-P, Schloegel M, Thibaud D and Bergamaschi R (1986) Colorectal cancer in patients under 40 years of age. *Dis Colon Rect* **29**: 322–325

Akhurst RJ and Balmain A (1999) Genetic events and the role of TGF beta in epithelial tumour progression. *J Pathol* **187**: 82–90

Alawi MA, Ammari N and Shuraiki Y (1992) Organochlorine pesticide contaminations in human milk samples from women living in Amman, Jordan. *Arch Env Contamin Toxicol* **23**: 235–239

Al-Jaberi TM, Ammari F, Gharieybeh K, Khammash M, Yaghan RJ, Heis H, Al-Omari M and Al-Omari N (1997) Colorectal adenocarcinoma in a defined Jordanian population from 1990 to 1995. *Dis Colon Rect* **40**: 1089–1094

Al-Saleh I, Echeverria-Quevedo A, Al-Dgaither S and Faris R (1998) Residue levels of organochlorinated insecticides in breast milk: a preliminary report from Al-Kharj, Saudi Arabia. *J Env Path Toxicol Oncol* **17**: 37–50

Anderberg MR (1973) *Cluster Analysis for Applications*. Academic Press, New York

Baas IO, Mulder JW, Offerhaus GJ, Vogelstein B and Hamilton SR (1994) An evaluation of six antibodies for immunohistochemistry of mutant p53 gene product in archival colorectal neoplasms. *J Pathol* **172**: 5–12

Bedikian AY, Kantarjian H, Nelson RS, Stroehlein JR and Bodey GP (1981) Colorectal cancer in young adults. *South Med J* **74**: 920–924

Boland CR, Thibodeau SN, Hamilton SR, Sidransky D, Eshleman JR, Burt RW, Meltzer SJ, Rodriguez-Bigas MA, Fodde R, Ranzani GN and Srivastava S (1998) A National Cancer Institute workshop on microsatellite instability for cancer detection and familial predisposition: development of international criteria for the determination of microsatellite instability in colorectal cancer. *Cancer Res* **58**: 5248–5257

Boytchev H, Marcovic S and Oettle GJ (1999) The characteristics of large bowel cancer in the low-risk population of the Witwatersrand. *J Royal Coll Surg Edin* **44**: 366–370

Breivik J, Lothe RA, Meling GI, Rognum TO, Borresen-Dale AL and Gaudernack G (1997) Different genetic pathways to proximal and distal colorectal cancer influenced by sex related factors. *Int J Cancer* **74**: 664–669

Calin GA, Gafa R, Tibileti MG, Herlea V, Becheanu G, Cavazzini L, Barbanti-Brodano G, Nenci I, Negrini M and Lanza G (2000) Genetic progression in microsatellite instability high (MSI-H) colon cancers correlates with clinico-pathological parameters: A study of the TGRbetaRII, BAX, hMSH3, hMSH6, IGFIIR and BLM genes. *Int J Cancer* **89**: 230–235

Chan TL, Yuen ST, Chung LP, Ho JWC, Kwan ASY, Ho JCY, Leung SY and Wyllie AH (1999) Frequent microsatellite instability and mismatch repair gene mutations in young Chinese patients with colorectal cancer. *J Natl Cancer Inst* **91**: 1221–1226

Chen MC, Chang PY, Chuang CY, Chen YJ, Wang FP, Tang YC and Chou SC (1981) Colorectal cancer and schistosomiasis. *Lancet* **1**: 971–973

Cox LS (1997) Multiple pathways control cell growth and transformation: Overlapping and independent activities of p53 and $p21^{Cip1/WAF1/Sdi1}$. *J Pathol* **183**: 134–140

Curtale F, Nabil M, El-Wakeel A and Shamy MY (1998) Anemia and intestinal parasitic infections among school age children in Behera Governorate, Egypt. *J Trop Ped* **44**: 323–328

Dietmaier W, Wallinger S, Bocker T, Kullmann F, Fishel R and Ruschoff J (1997) Diagnostic microsatellite instability: definition and correlation with mismatch repair protein expression. *Cancer Res* **57**: 5248–5257

Dieumegard B, Grandjouan S, Sabourin J-C, Le Bihan M-L, Lefrere I, Bellefqih, Pignon J-P, Rougier P, Lasser P, Benard J, Couturier D and Bressac-de Paillerets B (2000) Extensive molecular screening for hereditary non-polyposis colorectal cancer. *Br J Cancer* **82**: 871–880

Ding HF and Fisher DE (1998) Mechanisms of p53-mediated apoptosis. *Crit Rev Oncol* **9**: 83–98

El-Khoby T, Galal N, Fenwick A, Barakat R, El-Hawey A, Nooman Z, Habib M, Abdel-Wahab F, Gabr NS, Hammam HM, Hussein MH, Mikhail NN, Cline BL and Strickland GT (2000) The epidemiology of schistosomiasis in Egypt: Summary findings in nine governorates. *Am J Trop Med Hyg* **62**(2 Suppl): 88–89

Forster S, Sattler HP, Hack M, Romanakis K, Rohde V, Seitz G and Wullich B (1998) Microsatellite instability in sporadic carcinomas of the proximal colon: association with diploid DNA content, negative protein expression of p53, and distinct histopathologic features. *Surgery* **123**: 13–18

Fujiwara T, Stolker JM, Watanabe T, Rashid A, Longo P, Eshleman JR, Booker S, Lynch HT, Jass JR, Green JS, Kim H, Jen J, Vogelstein B and Hamilton SR (1998) Accumulated clonal genetic alterations in familial and sporadic colorectal carcinomas with widespread instability in microsatellite sequences. *Am J Pathol* **153**: 1063–1078

Genuardi M, Anti M, Capozzi E, Leonardi F, Fornasarig M, Novella E, Bellacosa A, Valenti A, Gabarrini GB, Roncucci L, Benatti P, Percesepe A, Ponz de Leon M, Coco C, de Paoli A, Valentini M, Boiocchi M, Neri G and Viel A (1998) MLH1 and MSH2 constitutional mutations in colorectal cancer families not meeting the standard criteria for hereditary nonpolyposis colorectal cancer. *Int J Cancer* **75**: 835–839

Greenlee RT, Murray T, Bolden S and Wingo PA (2000) Cancer Statistics, 2000. *CA Cancer J* **49**: 8–31

Gryfe R, Swallow C, Bapat B, Redston M, Gallinger S and Couture J (1997) Molecular biology of colorectal cancer. *Curr Probl Cancer* **21**: 233–300

Hainaut P, Hernandez T, Robinson A, Rodriguez-Tome P, Floures T, Holstein M, Harris CC and Montesano R (1998) IARC database of p53 mutations in human tumors on cell lines: Update compilation, revised formats and new visualization tools. *Nucleic Acids Res* **26**: 205–213

Herman JG, Graff JR, Myohanen S, Nelkin BD and Baylin SB (1996) Methylation-specific PCR: a novel PCR assay for methylation status of CpG islands. *Proc Natl Acad Sci USA* **93**: 9821–9826

Herman JG, Umar A, Polyak K, Graff JR, Ahuja N, Issa JP, Markowitz S, Willson JK, Hamilton SR, Kinzler KW, Kane MF, Kolodner RD, Vogelstein B, Kunkel TA and Baylin SB (1998) Incidence and functional consequences of hMLH1 promoter hypermethylation in colorectal carcinoma. *Proc Natl Acad Sci USA* **95**: 6870–6875

Heys SD, O'Hanrahan TJ, Brittenden J and Eremin O (1994) Colorectal cancer in young patients: a review of the literature. *Eur J Surg Oncol* **20**: 225–231

Iino H, Jass JR, Simms LA, Young J, Leggett B, Ajioka Y and Watanabe H (1999) DNA microsatellite instability in hyperplastic polyps, serrated adenomas, and mixed polyps: A mild mutator pathway for colorectal cancer? *J Clin Pathol* **52**: 5–9

Ilyas M, Straub J, Tomlinson IP and Bodmer WF (1999) Genetic pathways in colorectal and other cancers. *Eur J Cancer* **35**: 1986–2002

International Agency for Research on Cancer (1994) Schistosomes, Liver Flukes and Helicobacter pylori. *IARC Monog Eval Carcin Risk Hum* **61**: 68–100

 © 2001 Cancer Research Campaign

Ionov Y, Peinado MA, Malkhosyan S, Shibata D and Perucho M (1993) Ubiquitous somatic mutations in simple repeated sequences reveal a new mechanism for colonic carcinognesis. *Nature* **363**: 558–561

Isbister W (1992) Colorectal cancer below age 40 in the Kingdom of Saudi Arabia. *Aust NZJ Surg* **62**: 468–472

Jass JR (1995) Colorectal adenoma progression and genetic change: Is there a link? *Ann Med* **27**: 301–306

Jass JR, Biden KG, Cummings MC, Simms LA, Walsh M, Schoch E, Meltzer SJ, Wright C, Searle J, Young J and Leggett BA (1999) Characterizations of a subtype of colorectal cancer combining features of the suppressor and mild mutator pathways. *J Clin Pathol* **52**: 455–460

Jen J, Kim H, Piantadosi S, Liu Z-F, Levitt RC, Sistonen P, Kinzler KW, Vogelstein B and Hamilton SR (1994a) Allelic loss of chromosome 18q and prognosis of colorectal cancer. *N Engl J Med* **331**: 213–221

Jen J, Powell SM, Papadopoulos N, Smith KJ, Hamilton SR, Vogelstein B and Kinzler KW (1994b) Molecular determinants of dysplasia in colorectal lesions *Cancer Res* **54**: 5523–5526

Jiricny J (1998) Replication errors: Cha(lle)nging the genome. *EMBO J* **17**: 6427–6436

Kane MF, Loda M, Gaida GM, Lipman J, Mishra R, Goldman H, Jessup JM and Kolodner R (1997) Methylation of the hMLH1 promoter correlates with lack of expression of hMLH1 in sporadic colon tumors and the mismatch repair-defective human tumor cell lines. *Cancer Res* **57**: 808–811

Kim J, Jen J, Vogelstein B and Hamilton SR (1994) Clinical and pathological characteristics of sporadic colorectal carcinomas with DNA replication errors in microsatellite sequences. *Am J Pathol* **145**: 148–156

Kinzler KW and Vogelstein B (1996) Lessons from hereditary colorectal cancer. *Cell* **87**: 159–170

Konishi M, Kikuchi-Yanoshita R, Tanaka K, Muraoka M, Onda A, Okumura Y, Kishi N, Iwama T, Mori T, Koike M, Ushio K, Chiba M, Nomizu S, Konishi F, Utsunomiya J and Miyaki M (1996) Molecular nature of colon tumors in hereditary nonpolyposis colon cancer, familial polyposis, and sporadic colon cancer. *Gastroenterology* **111**: 307–317

Kuismanen SA, Holmberg MT, Salovaara R, de la Chapelle A and Peltomaki P (2000) Genetic and epigenetic modification of MLH1 accounts for a major share of microsatellite-unstable colorectal cancers. *Am J Pathol* **156**: 1773–1779

Lengauer C, Kinzler KW and Vogelstein B (1998) Genetic instabilities in human cancers. *Nature* **396**: 643–649

Levine AJ (1997) p53, the cellular gatekeeper for growth and division. *Cell* **88**: 323–331

Liu B, Farrington SM, Petersen GM, Hamilton SR, Parsons R, Papadopoulos N Fujiwara T, Jen J, Kinzler KW and Wyllie AH (1995) Genetic instability occurs in the majority of young patients with colorectal cancer. *Nat Med* **1**: 348–352

Losi L, Ponz de Leon M, Jiricny J, Di Gregorio C, Benatti P, Percesepe A, Fante R, Roncucci L, Pedroni M and Benhattar J (1997) K-ras and p53 mutations in hereditary non-polyposis colorectal cancers. *Int J Cancer* **74**: 94–96

Lukish JR, Muro K, DeNobile J, Katz R, Williams J, Cruess DF, Drucker W, Kirsch I and Hamilton SR (1998) Prognostic significance of DNA replication errors in young patients with colorectal cancer. *Ann Surg* **227**: 51–56

Lynch HT and Lynch JF (2000) Hereditary nonpolyposis colorectal cancer. *Semin Surg Oncol* **18**: 305–313

Magrath I and Litvak J (1993) Cancer in developing countries: opportunities and challenge. *J Natl Cancer Inst* **85**: 862–874

Moskaluk CA, Heitmiller R, Zahurak M, Schwab B, Sidransky D and Hamilton SR (1996) p53 and p21$^{WAF1/CIP1/SD11}$ gene products in Barrett esophagus and adenocarcinoma of the esophagus and esophago gastric junction. *Human Pathol* **27**: 1211–1220

Moskaluk CA and Kern SE (1997) Microdissection and polymerase chain reaction amplification of genomic DNA from histological tissue sections. *Am J Pathol* **150**: 1547–1552

Muller W, Burgart LJ, Krause-Paulus R, Thibodeau SN, Almeida M, Edmonston TB, Boland CR, Sutter C, Jass JR, Lindblom A, Lubinski J, MacDermot K, Sanders DSA, Morreau H, Muller A, Oliani C, Orntoft T, Ponz De Leon M, Rosty C, Rodgriguez-Bigas M, Ruschoff J, Ruszkiewicz A, Sabourin J, Salovaara R, Moslein G and ICG-HNPCC (International Collaborative Group) (accepted for publication) The value of immunohistochemistry in the diagnosis of hereditary nonpolyposis colorectal cancer (HNPCC) – Results of an international collaborative study. *Familial Cancer*

Nucci MR, Robinson CR, Longo P, Campbell P and Hamilton SR (1997) Phenotypic and genotypic characteristics of aberrant crypt foci in human colorectal mucosa. *Human Pathol* **28**: 1396–1407

Olschwang S, Hamelin R, Laurent-Puig P, Thuille B, DeRycke Y, Li Y-J, Muzeau F, Girodet J, Salmon RJ and Thomas G (1997) Alternative genetic pathways in colorectal carcinogenesis. *Proc Natl Acad Sci USA* **94**: 12122–12127

Parkin DM, Muir CS, Whelan SL, Gao YT, Ferlay J and Powell J (eds.) (1992) Cancer incidence in five continents. *IARC Scientific Publ* **120**: 182–185

Parsons R, Myeroff LL, Liu B, Willson JK, Markowitz SD, Kinzler KW and Vogelstein B (1995) Microsatellite instability and mutations of the transforming growth factor beta type II receptor gene in colorectal cancer. *Cancer Res* **55**: 5548–5550

Perucho M (1999) Correspondence re: Boland CR et al. A National Cancer Institute workshop on microsatellite instability for cancer detection and familial predisposition: development of international criteria for the determination of microsatellite instability in colorectal cancer (*Cancer Res* **58**: 5248–5257, 1998). *Cancer Res* **59**: 249–253

Peters RK, Garabant DH, Yu MC and Mack TM (1989) A case-control study of occupational and dietary factors in colorectal cancer in young men by subsite. *Cancer Res* **49**: 5459–5468

Prolla TA (1998) DNA mismatch repair and cancer. *Curr Opin Cell Biol* **10**: 311–316

Rembacken BJ, Fuji T, Cairns A, Dixon MF, Yoshida S, Chalmers DM and Axon AT (2000) Flat and depressed colonic neoplasms: A prospective study of 1000 colonoscopies in the UK. *Lancet* **355**: 1211–1214

Richter ED and Safi J (1997) Pesticide use, exposure, and risk: a joint Israeli-Palestinian perspective. *Environment Res* **73**: 211–218

Rockhill B and Giovannucci E (1999) Editorial. Cancer mortality rates in Menofeia, Egypt: comparison with US mortality rates. *Cancer Causes and Control* **10**: 345–347

Rubin BM, Doman DB, Goldberg HJ and Golding MI (1998) Colon cancer in young persons of East African descent. *Am J Gastroenterol* **93**: 1014–1015

Ruschoff J, Dietmaier W, Luttges J, Seitz G, Bocker T, Zirngibl H, Schlegel J, Schackert HK, Jauch KW and Hofstaedter F (1997) Poorly differentiated adenocarcinoma, medullary type: clinical, phenotypic, and molecular characteristics. *Am J Pathol* **150**: 1815–1825

Servomaa K, Kiuru A, Kosma V-M, Hirvikoski P and Rytomaa T (2000) p53 and K-ras gene mutations in carcinoma of the rectum among Finnish women. *J Clin Pathol Mol Pathol* **53**: 24–30

Soliman AS, Bondy ML, Levin B, Hamza MR, Ismail K, Ismail S, Hammam HM, el-Hattab OH, Kamal SM, Soliman AG, Dorgham LA, McPherson RS and Beasley RP (1997a) Colorectal cancer in Egyptian patients under 40 years of age. *Int J Cancer* **71**: 26–30

Soliman AS, Smith MA, Cooper SP, Ismail K, Khaled H, Ismail S, McPherson RS, Seifeldin IA and Bondy ML (1997b) Serum organochlorine pesticide levels in patients with colorectal cancer in Egypt. *Arch Environ Health* **52**: 409–415

Soliman AS, Bondy ML, Guan Y, El-Badawi S, Mokhtar N, Bayomi S, Raouf AA, Ismail S, McPherson RS, Abdel-Hakim TF, Beasley RP, Levin B and Wei Q (1998a) Reduced expression of mismatch repair genes in colorectal cancer patients in Egypt. *Int J Oncol* **12**: 1315–1319

Soliman AS, Bondy ML, Levin B, El-Badawy S, Khaled H, Hablas A, Ismail S, Adley M, Mahgoub KG, McPherson RS and Beasley RP (1998b) Familial aggregation of colorectal cancer in Egypt. *Int J Cancer* **77**: 811–816

Soliman AS, Bondy ML, Raouf AA, Makram MA, Johnston DA and Levin B (1999) Cancer mortality in Menofeia, Egypt: comparison with U.S. mortality rates. *Cancer Causes and Control* **10**: 349–354

Statistical Analysis System User's Guide: Basics, Version 6.0. (1990) Cary, North Carolina: Statistical Analysis System Institute, Inc.

Steele GD Jr. (1994) The National Cancer Data Base report on colorectal cancer. *Cancer* **74**: 1979–1989

Tang W-Y, Elnatan J Lee Y-S, Goh H-S and Smith DR (1999) c-Ki-*ras* mutations in colorectal adenocarcinomas from a country with a rapidly changing colorectal cancer incidence. *Br J Cancer* **81**: 237–241

Temtamy SA, Kandil MR, Demerdash AM, Hassan WA, Meguid NA and Afifi, HH (1994) An epidemiological/genetic study of mental subnormality in Assiut Governorate, Egypt. *Clin Genet* **46**: 347–351

Umetani N, Sasaki S, Watanabe T, Shinozaki M, Matsuda K, Ishigami H, Ueda E and Muto T (1999) Genetic alterations in ulcerative colitis-associated neoplasia focusing on APC, K-ras gene and microsatellite instability. *Jpn J Cancer Res* **90**: 1081–1087

Vasen HFA, Mecklin JP, Kahn PM and Lynch HT (1991) Hereditary non-polyposis colorectal cancer. *Lancet* **338**: 877

Weisburger JH, Reddy BS and Spingarn NE (1980) Current views on the mechanisms involved in the etiology of colorectal cancer. In *Colorectal Cancer: Prevention, Epidemiology, and Screening*, S. Winawer, D. Schottenfeld, and P. Sherlock (eds.) pp. 19–41. Raven Press: New York

*© 2001 Cancer Research Campaign*

Wijnen J, Khan PM, Vasen H, van der Klift H, Mulder A, van Leeuwen-Cornelisse I, Bakker B, Losekoot M, Moller P and Fodde R (1997) Hereditary nonpolyposis colorectal cancer families not complying with the Amsterdam criteria show extremely low frequency of mismatch repair gene mutations. *Am J Hum Genet* **61**: 329–335

Williams PR, Stegens NL and Goldsmith JR (1977) Associations of cancer site and type with occupation and industry from the Third National Cancer Survey Interview. *J Natl Cancer Inst* **59**: 1147–1185

Yagi OK, Akiyama Y, Nomizu T, Iwama T, Endo M and Yuasa Y (1998) Proapoptotic gene BAX is frequently mutated in hereditary nonpolyposis colorectal cancer but not in adenomas. *Gastroenterology* **114**: 268–274

Zhang R, Takahashi S, Orita S, Yoshida A, Maruyama H, Shirai T and Ohta N (1998) p53 gene mutations in rectal cancer associated with schistosomiasis japonica in Chinese patients. *Cancer Lett* **131**: 215–221

*© 2001 Cancer Research Campaign*
